# Postsynaptic Ca_V_1.1-driven calcium signaling coordinates presynaptic differentiation at the developing neuromuscular junction

**DOI:** 10.1038/s41598-019-54900-w

**Published:** 2019-12-05

**Authors:** Mehmet Mahsum Kaplan, Bernhard E. Flucher

**Affiliations:** Department of Physiology and Medical Physics, Medical University Innsbruck, 6020 Innsbruck, Austria

**Keywords:** Synaptic development, Ion channels in the nervous system

## Abstract

Proper formation of neuromuscular synapses requires the reciprocal communication between motor neurons and muscle cells. Several anterograde and retrograde signals involved in neuromuscular junction formation are known. However the postsynaptic mechanisms regulating presynaptic differentiation are still incompletely understood. Here we report that the skeletal muscle calcium channel (Ca_V_1.1) is required for motor nerve differentiation and that the mechanism by which Ca_V_1.1 controls presynaptic differentiation utilizes activity-dependent calcium signaling in muscle. In mice lacking Ca_V_1.1 or Ca_V_1.1-driven calcium signaling motor nerves are ectopically located and aberrantly defasciculated. Axons fail to recognize their postsynaptic target structures and synaptic vesicles and active zones fail to correctly accumulate at the nerve terminals opposite AChR clusters. These presynaptic defects are independent of aberrant AChR patterning and more sensitive to deficient calcium signals. Thus, our results identify Ca_V_1.1-driven calcium signaling in muscle as a major regulator coordinating multiple aspects of presynaptic differentiation at the neuromuscular synapse.

## Introduction

The development of the neuromuscular junction (NMJ) critically depends on tightly regulated trans-synaptic mechanisms^1^. During NMJ formation, muscle fibers are intrinsically pre-specialized by clustering postsynaptic proteins, such as MuSK^2^ and acetylcholine receptors (AChRs)^3–5^, in the prospective central synaptic region to where motor axons are targeted for innervation. Upon nerve arrival the nerve-derived factor agrin and its postsynaptic receptor LRP4/MuSK stabilize and maturate AChR clusters at the synaptic region, whereas secretion of the neurotransmitter ACh extinguishes extrasynaptic AChRs by counteracting the agrin/LRP4/MuSK pathway^6–11^. Recently we demonstrated that AChR patterning in the center of the muscle fibers is controlled by calcium signals initiated by Ca_V_1.1, a voltage-gated calcium channel and voltage-sensor for skeletal muscle excitation-contraction (EC) coupling^12^. This postsynaptic defect was accompanied by aberrant growth patterns of the motor nerves. However, whether this presynaptic phenotype was simply caused by the redistribution of its postsynaptic targets or by AChR/MuSK-independent mechanisms remained unanswered.

On the presynaptic side differentiation of the motor axons involves multiple characteristic steps, including the growth and branching pattern of motor nerve fascicles in the center of the muscle, the recognition of the postsynaptic target sites, and the differentiation of the nerve terminals opposite the AChR clusters. Each of these steps is regulated by specific retrograde signaling mechanisms originating from the muscle^13^. Correct fasciculation of the ingrowing motor nerves is regulated by muscle β-catenin and Slit2/Robo signaling^14–16^. LRP4, β1-integrin, MuSK and Dok7 are required for motor axons to recognize their termination territory and/or their postsynaptic target structures^6,17–21^. Upon establishing the synaptic contacts, presynaptic proteins become concentrated at the axon terminals facing the postsynaptic AChR clusters, a process regulated by FGF7/10/22^22^ and NCAM^23^. These findings indicate that the comprehensive differentiation of the motor axons requires complex and temporally coordinated regulation by multiple trans-synaptic mechanisms. The mechanisms regulating these signals in the postsynaptic muscle cell are still poorly understood and the possible role of postsynaptic calcium signals is unknown. However, similar presynaptic defects noticed in mouse mutants lacking synaptic transmission indicates an involvement of activity-regulated mechanisms^10,24^.

In skeletal muscle electrical activity initiated at the NMJs is translated into muscle contraction by a process called EC coupling, which is under the control of Ca_V_1.1^25^. In adult muscle, Ca_V_1.1 acts solely as a voltage-sensor to mechanically translate muscle action potentials into activation of ryanodine receptors (RyR1), which, in response, mobilize calcium from intracellular stores^25–29^. In embryonic muscles, exclusion of exon 29 in Ca_V_1.1 gives rise to a channel splice variant (Ca_V_1.1e) with increased open probability and voltage-sensitivity^30–32^. The activity-dependent calcium influx through this embryonic Ca_V_1.1 splice variant is sufficient to support the normal patterning of AChR clusters in the center of muscle fibers^12^.

In mutant mouse models lacking Ca_V_1.1-driven calcium signals, the central patterning of AChR clusters fails and the muscles become hyper-innervated^12,33^. Here we demonstrate that the aberrant growth and innervation of the motor axons in the calcium channel mutants is not limited to the extent expected from the wider distribution of their target structures, but include characteristic properties established before and after contacting AChR clusters. To dissect these individual aspects of motor nerve differentiation and examine their potential dependence on Ca_V_1.1-driven calcium signals, we analyzed nerve growth patterns, fasciculation, target recognition, and differentiation of nerve terminals in three genetic mouse models. Our results demonstrate that (1) all the aspects of presynaptic nerve growth and differentiation listed above are defective in mice lacking Ca_V_1.1 or Ca_V_1.1-driven calcium signals, (2) the motor nerve defects occur independently of aberrant AChR patterning, and (3) the presynaptic motor nerve defects show a stronger dependence on muscle calcium signals than the postsynaptic AChR patterning defects. Thus, the scope and extent of presynaptic aberrations in these mice indicate a central role of postsynaptic Ca_V_1.1-driven calcium signals in regulating presynaptic differentiation at the developing NMJ.

## Results

### Ca_V_1.1-driven calcium signaling regulates motor nerve positioning and projections

**Figure 1:**
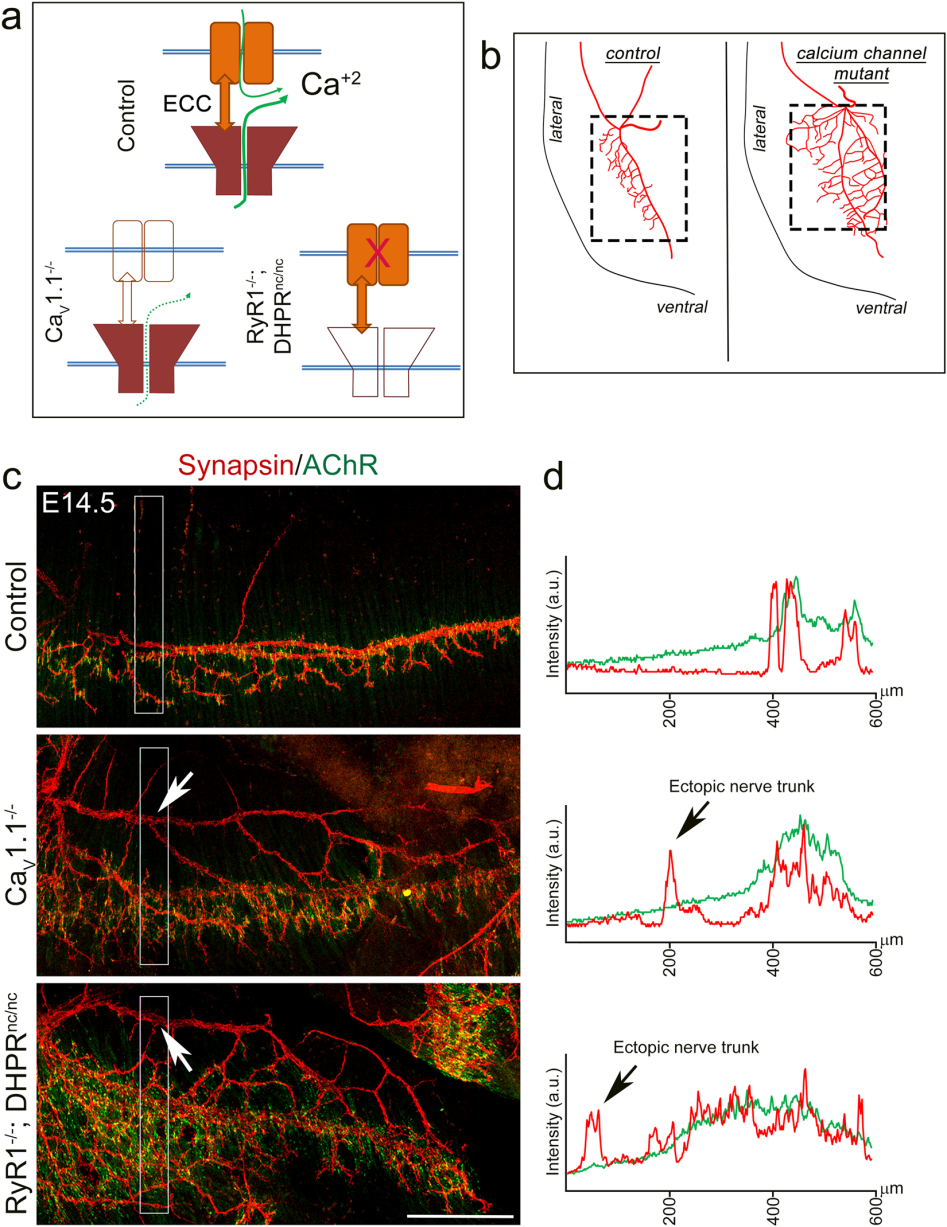
The lack of Ca_V_1.1-driven calcium signaling leads to aberrant motor nerve positioning and projections. **(a)** Illustration of the calcium signals generated by the interaction between Ca_V_1.1 and RyR1 in skeletal muscle ECC (excitation-contraction coupling) and the lack of Ca_V_1.1-driven calcium signaling in Ca_V_1.1^−/−^ and RyR1^−/−^; DHPR^nc/nc^ mice. Ca_V_1.1-independent calcium leak through uncoupled RyR1 in Ca_V_1.1^−/−^ mice is indicated by a dashed green arrow. **(b)** Schematic illustration of aberrant nerve positioning and projections and the location of the analyzed region (dashed frame) in the left ventral quadrant of the mouse diaphragm in control and calcium channel mutant mice. **(c)** Representative fluorescence micrographs of left-ventral diaphragms from E14.5 control, Ca_V_1.1^−/−^, and RyR1^−/−^; DHPR^nc/nc^ mice. Motor nerve branches are labeled with anti-synapsin (red) and AChRs with α-BTX (green). Scale bar: 300 μm. **(d)** Linescan fluorescence intensity blots of the band indicated by the white boxes in **(c)**.

Homozygous Ca_V_1.1^−/−^ (dysgenic) mice lack the voltage-sensor for EC coupling as well as the L-type calcium currents (LTCC) prominent in embryonic muscles^25,30^. In these mice the calcium release channel (RyR1) is present but no longer activated in response to membrane depolarization; yet it displays increased calcium leak^34^. RyR1^−/−^; DHPR^nc/nc^ double-knockout mice lack the calcium release channel and express a non-conducting variant of Ca_V_1.1. Although a functional voltage-sensor is expressed, both activity-dependent calcium sources—LTCCs and calcium release from the sarcoplasmic reticulum—are lacking^12,35–37^ (Fig. 1a). Analysis of NMJ formation in diaphragm muscles at embryonic day 14.5 to shortly before birth (E14.5–E18.5) indicated striking abnormalities in the growth patterns and differentiation of the presynaptic motor nerve.

Motor axons navigate through muscle fibers in bundles and stereotypically branch during innervation of the diaphragm muscle. The mechanisms that control this characteristic branching pattern are largely unknown. In the ventral part of the left half-diaphragm of wildtype mice at embryonic day 14 (E14.5), a single nerve trunk is located near the center of the muscle. Short secondary branches extend from it mostly towards the lateral direction and innervate the AChR clusters located in a narrow endplate band in the center of the diaphragm muscle (Fig. 1b–d). In contrast, in *dysgenic* Ca_V_1.1^−/−^ and in RyR1^−/−^; DHPR^nc/nc^ diaphragms stained with anti-synapsin and α-BTX at E14.5, the arriving motor nerves displayed aberrant projections. The main central nerve trunk extended longer secondary branches, which often formed extensive axon networks. In addition to the central nerve trunk, an ectopic trunk emerged from the phrenic nerve at the entry point into the left diaphragm. This branch failed to be targeted to the center of the diaphragm but consistently remained located in the periphery of the muscle, near the ventral tendinous cavity. From there it projected secondary branches toward the central muscle region where they coalesced with the branches in the muscle center and formed an extensive network on the lateral side of the diaphragm muscle (Fig. 1b–d).

These motor nerve defects equally occur in Ca_V_1.1^−/−^ mice, which lack the LTCCs and the voltage-sensor for EC coupling, and in the RyR1^−/−^; DHPR^nc/nc^ double mutant mice, which express the voltage sensor, but lack calcium influx and release. Therefore a possible involvement of a calcium channel-independent role of the Ca_V_1.1 protein can be excluded. Rather, this observation in both calcium channel mutants indicates a critical role of muscle calcium signaling in the retrograde regulation of nerve positioning and the projection pattern.

### Ca_V_1.1-driven calcium signaling in muscle regulates the fasciculation of the motor nerves

**Figure 2:**
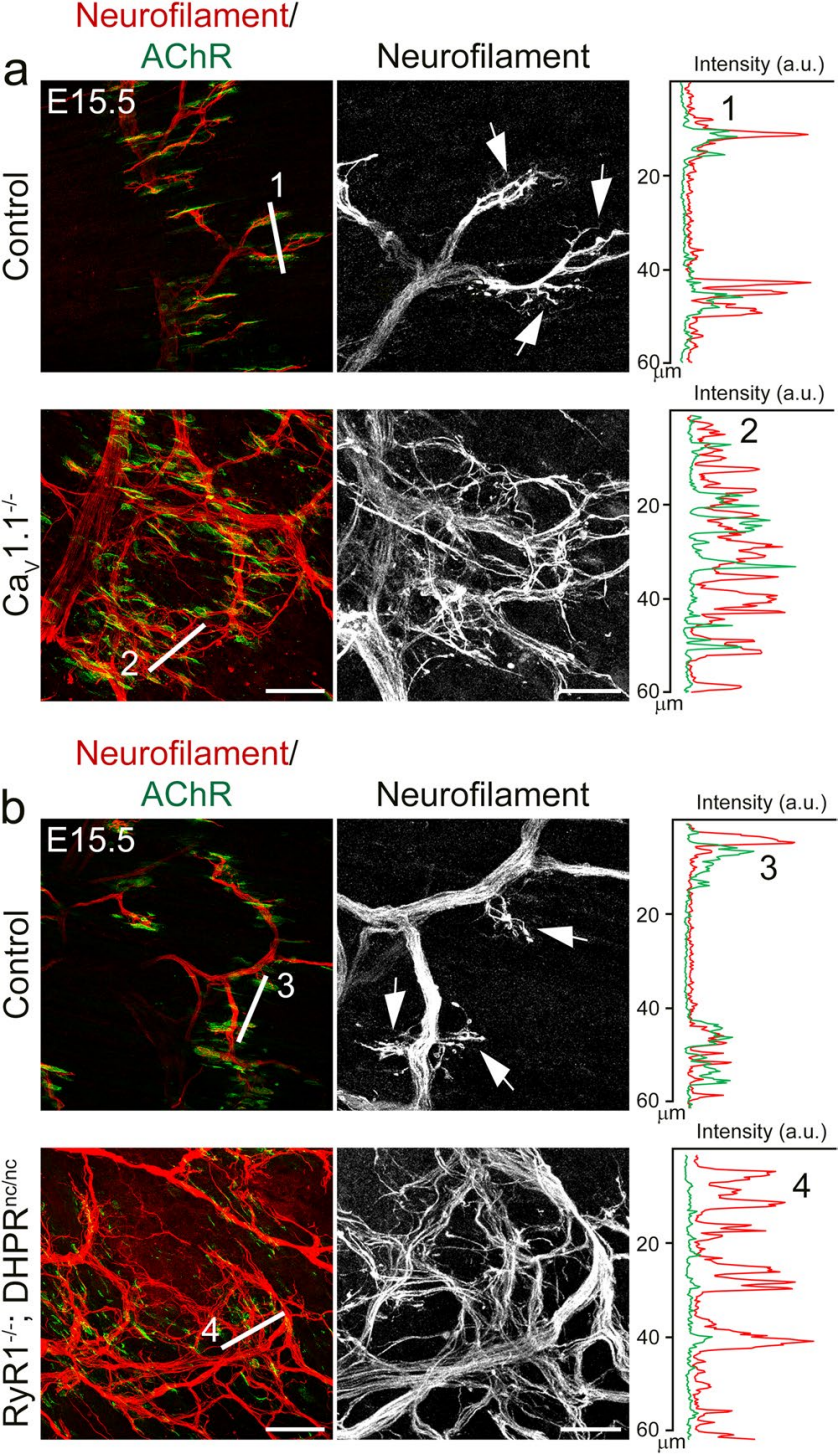
Ca_V_1.1-driven calcium signaling is essential for correct motor nerve fasciculation. **(a,b)** Double-labeling of motor axons and AChRs with neurofilament antibody (red) and α-BTX (green), respectively, in E15.5 diaphragms from control, Ca_V_1.1^−/−^, and RyR1^−/−^; DHPR^nc/nc^ mice. Axon defasciculation (examples indicated by arrows) in controls is restricted to the terminal branches at the AChR clusters, whereas axons unrestrictedly separate from each other in Ca_V_1.1^−/−^ and RyR1^−/−^; DHPR^nc/nc^ mice. The linescans show the fluorescence intensity along the lines (60 μm) in the color images and demonstrate that coincidence of terminal axon branches with postsynaptic AChR clusters in controls, but not in mutant diaphragms. Scale bar: 50 μm. Motor axons are displayed in zoomed images on the right. Scale bar: 20 μm.

Peripherally located nerve trunks in embryonic mouse diaphragm muscles, as observed in the calcium channel mutants (above), are commonly associated with aberrant defasciculation of the nerves. This has previously been described in mice with targeted mutations in the CLP1, Slit2, Robo1/2, and β-catenin genes^14–16,38^. To examine whether a similar concurrence of phenotypes is also the case in the two muscle calcium channel mutants, we stained E14.5 (Suppl. Fig. 1) and E15.5 (Fig. 2) diaphragm muscles with neurofilament antibody and α-BTX and analyzed motor axon bundles. The examples shown in Fig. 2 demonstrate that in controls the motor axons were densely bundled in the primary and secondary branches. In controls defasciculation occurred exclusively at the terminal branches where the motor neurons approached the synaptic boutons, as indicated by the α-BTX-stained postsynaptic AChR clusters. In contrast, in the absence of Ca_V_1.1-mediated calcium signaling in Ca_V_1.1^−/−^ and RyR1^−/−^; DHPR^nc/nc^ mice motor axons failed to stay in bundles, resulting in severely perturbed axon fasciculation at all levels of motor nerve branches. While some fasciculation still occurs many axons form loose networks apart of nerve bundles. Also along their length individual nerve bundles alternate between fasciculated and defaciculated segments. Whereas in control diaphragms defasciculation of the terminal branches closely corresponded to the location of postsynaptic AChR clusters (Fig. 2; line scans), in the two calcium channel mutants axon defasciculation appeared to be independent of their position relative to the AChR clusters. Observing these characteristic defasciculation defects in both calcium channel mutants as early as E14.5 (Suppl. Fig. 1) indicates that lacking activity-dependent calcium signaling in the muscle cells disrupts retrograde mechanisms responsible for the fasciculation of the motor neurons from the onset of NMJ development.

### Ca_V_1.1-mediated calcium signaling is required for correct motor axon navigation

**Figure 3:**
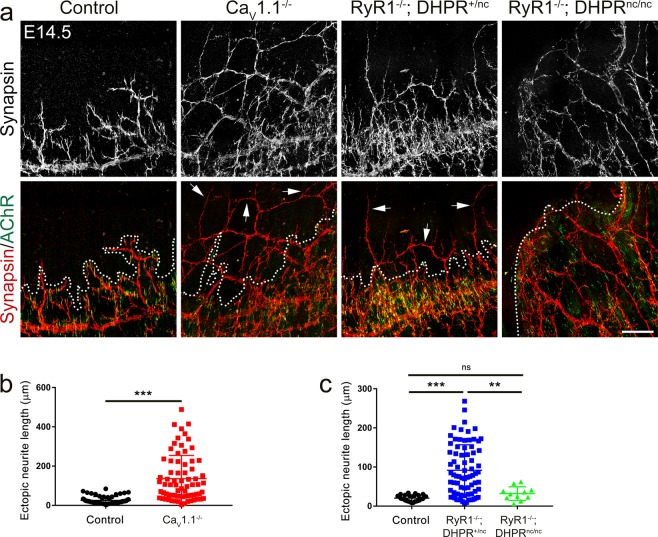
Ca_V_1.1-mediated calcium signals regulate motor axon navigation to the endplate band during early NMJ formation. **(a)** Double-labeling of motor nerve branches and AChRs with anti-synapsin (red) and α-BTX (green), respectively, in E14.5 diaphragms from control, Ca_V_1.1^−/−^, RyR1^−/−^; DHPR^+/nc^, and RyR1^−/−^; DHPR^nc/nc^ mice. Dashed lines deliniate the borders of the endplate bands in left dorsal diaphragms. Arrows indicate neurites traversing beyond the endplate band border. Scale bar: 100 μm. **(b,c)** Quantification of the neurite length beyond the endplate band. N ≥ 3 diaphragms from at least 3 litters for each genotype; mean ± sem; t-test for control vs Ca_V_1.1^−/−^ mice; one way ANOVA for control vs RyR1^−/−^; DHPR^+/nc^ vs RyR1^−/−^; DHPR^nc/nc^ mice: F(2, 112) = 20.23; p < 0.0001, Tukey’s multiple comparisons test: ns = 0.8186,,**p = 0.0025, ***p < 0.001.

During the onset of NMJ formation, motor axons are directed to the presumptive central synaptic zone for correct innervation of the muscle^39^. Whereas the nature of the guidance cues is elusive, it has been shown that local transcription of MuSK plays an important instructive role in this process, determining the territory in the postsynaptic muscle fibers where AChRs are clustered and motor axons grow to form synapses^2^. Because in Ca_V_1.1^−/−^ and RyR1^−/−^; DHPR^nc/nc^ mice the postsynaptic AChR clusters are not restricted to a central endplate band, it is not surprising to see the nerves project to a broader region of the diaphragm muscle. Nevertheless, if the hyper-innervation and branching phenotype were secondary to the AChR patterning defects, the border of the endplate band containing AChR clusters would be expected to also limit the axon growth, even if it is considerably widened as in Ca_V_1.1^−/−^ and RyR1^−/−^; DHPR^nc/nc^ mice^12^. This imaginary endplate band border can be traced by connecting the outermost located AChR clusters in the green channel of the double-immunofluorescence images (Fig. 3a, dashed line). After super-positioning the red channel showing synapsin-labeled nerve branches it can easily be assessed whether the axon branches remain confined to within this border or extend beyond it. As expected in wildtype mice at E14.5 motor axons did not grow beyond the endplate band (Fig. 3a, left column), indicating that ingrowing naïve neurites recognize the pre-specialized endplate band as their termination territory. However, this was not the case in calcium channel mutant mice. As reported earlier^12^ in Ca_V_1.1^−/−^ mice the AChR clusters were distributed in a wider region; but unexpectedly the motor axons did not respect this border and grew well beyond the widened endplate band (136.2 ± 13.8 μm axon branch length beyond endplate band in Ca_V_1.1^−/−^ mice compared to 25.02 ± 2.36 μm in wildtype; mean ± sem; N, ≥6 diaphragms and 62 sprouting branches, t-test p < 0.0001) (Fig. 3b). This indicates that Ca_V_1.1 is essential for directing arriving motor axons to the endplate band and that the excessive growth and branching of the motor axons in the Ca_V_1.1^−/−^ calcium channel mutant is not the immediate consequence of the increased number and wider distribution of the postsynaptic AChR clusters.

In order to test the importance of the magnitude of the Ca_V_1.1-mediated calcium signals in the regulation of motor nerve navigation, we analyzed mice in which Ca_V_1.1-driven calcium signals are totally ablated (RyR1^−/−^; DHPR^nc/nc^) and mice expressing LTCCs at half the current density (RyR1^−/−^; DHPR^+/nc^)^12,35^. Interestingly in RyR1^−/−^; DHPR^nc/nc^ mice the overall extent of motor axon growth correlated with the width of the endplate band. Here the postsynaptic AChR clusters were distributed widely almost across the entire width of the diaphragm muscle and a central accumulation of AChR clusters forming an endplate band was barely recognizable. Under these conditions growth of the axons beyond the limits of AChR clustering was no longer observed. However, the presence of LTCCs at half the current density in semi-heterozygous RyR1^−/−^; DHPR^+/nc^ mice largely rescued AChR patterning but not motor axon growth beyond the endplate (Fig. 3a,c). Thus, the complete ablation of LTCCs and SR calcium release leads to disruption of AChR patterning and axon outgrowth to the same extent, whereas in the presence of small calcium signals (leak through uncoupled RyR1 in Ca_V_1.1^−/−^ or LTCCs at half current density in RyR1^−/−^; DHPR^+/nc^)^34,35^ AChR patterning and motor axon growth are differentially affected. Whereas, some residual calcium signaling appears to be sufficient for the formation of a discernable endplate band of AChR clusters, this is not sufficient to prevent the axon growth beyond the borders of these endplate bands.

### Ca_V_1.1-mediated calcium signaling is required for the synaptic target recognition of motor axons

**Figure 4:**
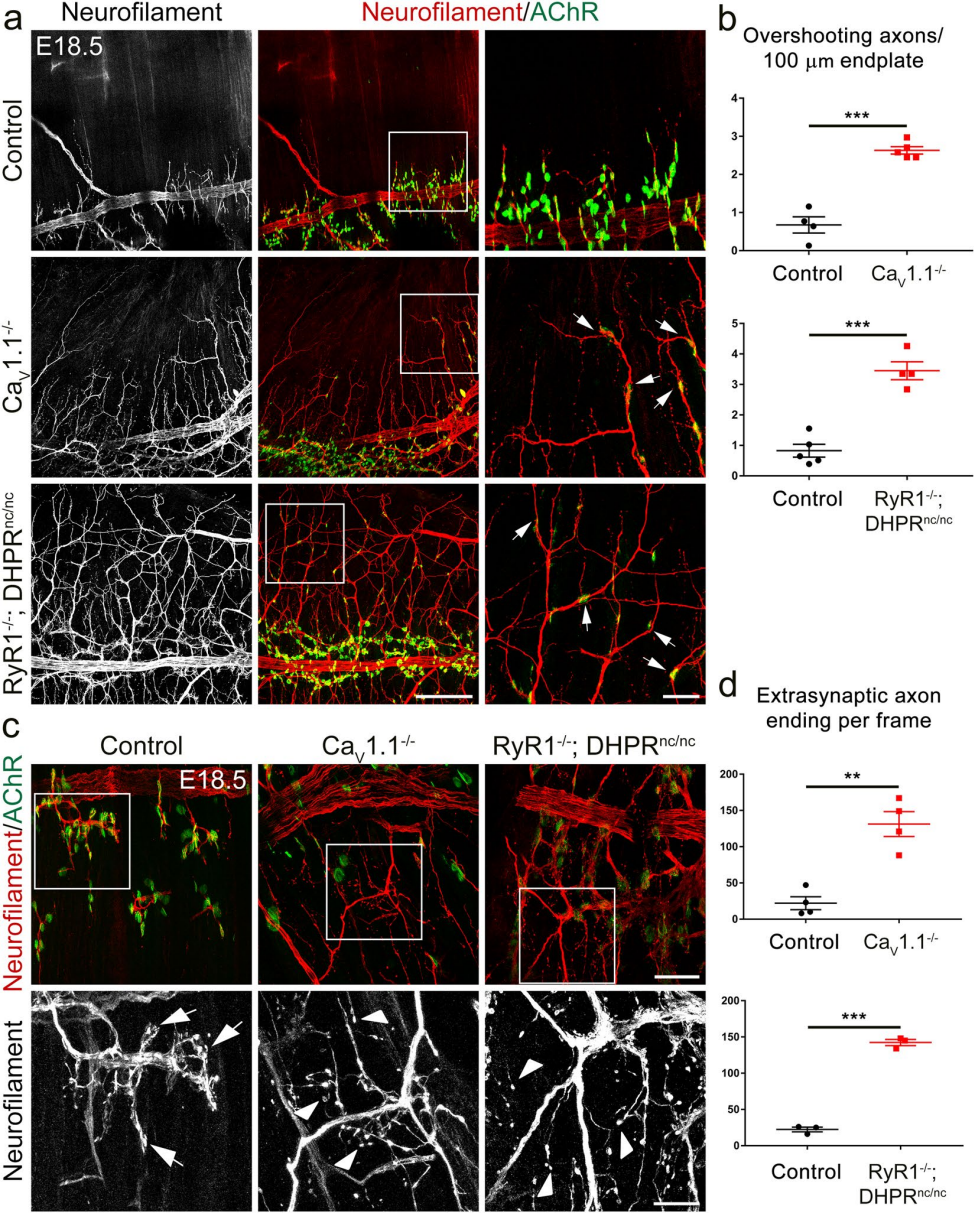
Synaptic target-recognition fails in muscles lacking Ca_V_1.1-mediated calcium signaling. **(a,c)** Motor axons and AChRs in E18.5 diaphragm muscles are stained with anti-neurofilament (red) and α-BTX (green), respectively. **(a)** In Ca_V_1.1^−/−^, and RyR1^−/−^; DHPR^nc/nc^ mice but not in controls, motor axons overshoot and fail to stop at the AChR clusters (examples indicated by arrows). Scale bar: 200 μm. Regions in white boxes are magnified in the micrographs at right. Scale bar: 50 μm. **(b)** Quantitative analysis of secondary nerve branches overshooting AChR clusters. N ≥ 4 diaphragms from at least 3 litters for each genotype; mean ± SEM; t-test, ***p < 0.0001. **(c)** Higher magnification images show axons terminate at AChR clusters in wildtype controls (examples shown by arrows), whereas in Ca_V_1.1^−/−^ and RyR1^−/−^; DHPR^nc/nc^ diaphragms growth cone-like axon endings (arrowheads) are randomly located in extrasynaptic domains. Scale bars: 50 μm; zoomed images below, 20 μm. **(d)** Quantitative analysis of axon endings not co-localized with AChR clusters. N ≥ 3 diaphragms from at least 3 litters for each genotype; mean ± SEM; t-test, **p = 0.0014, ***p < 0.0001.

Axon growth beyond the border of the endplate band at E14.5 suggested that in the calcium channel mutants the ingrowing axons failed to recognize their postsynaptic targets. To assess whether axon navigation defects persisted during development beyond E14.5 and to further examine the involvement of Ca_V_1.1-driven calcium in the regulation of target-recognition and motor axon termination upon contacting AChR clusters, we analyzed E18.5 Ca_V_1.1^−/−^ and RyR1^−/−^; DHPR^nc/nc^ diaphragms labeled with neurofilament antibody and α-BTX. We found that the lack of Ca_V_1.1 or Ca_V_1.1-mediated calcium signaling in these genotypes leads to severe axon termination and arborization defects. At E18.5 AChR clusters are considerably matured and are contacted by axons both in wildtype and in the two calcium channel mutants. In control mice motor axons typically terminated at postsynaptic AChR clusters, indicative of properly formed nerve-muscle synapses. In contrast motor axons in Ca_V_1.1^−/−^ or RyR1^−/−^; DHPR^nc/nc^ diaphragms frequently contacted AChR clusters *en passant*, however they apparently failed to collapse the growth cone and continued growing beyond the AChR clusters (Fig. 4a,b). Accordingly, in controls the positions of the motor axon endings typically correlated with the postsynaptic AChR clusters. However, in Ca_V_1.1^−/−^and RyR1^−/−^; DHPR^nc/nc^ diaphragms, axons ended in extrasynaptic muscle domains, and even at E18.5 displayed growth cones (Fig. 4c,d). Apparently, Ca_V_1.1-mediated calcium signals are required for the motor axons to recognize their postsynaptic targets, arrest their growth, and differentiate nerve terminals opposite to AChR clusters.

### Targeting of synaptic vesicle and active zone markers requires Ca_V_1.1-mediated calcium signaling

**Figure 5:**
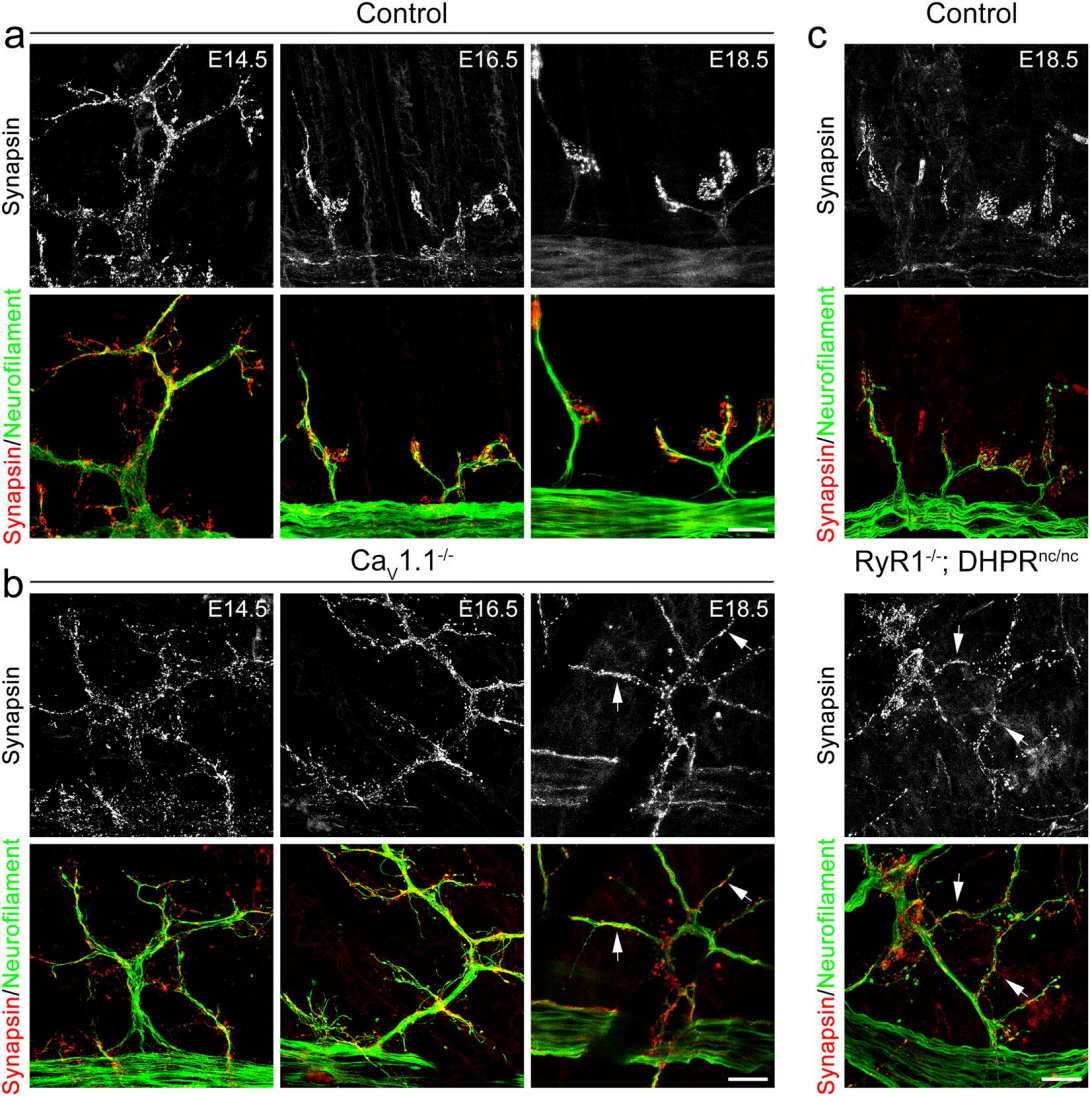
Synaptic vesicles stay distributed in the motor axons of mice lacking Ca_V_1.1 or Ca_V_1.1-mediated calcium signaling. **(a,b)** Synaptic vesicles and axons are double-labeled with anti-synapsin (red) and anti-neurofilament (green), respectively, in diaphragms from control **(a)** and from Ca_V_1.1^−/−^ mice **(b)** at E14.5, E16.5, and E18.5. During late fetal development synaptic vesicles effectively get concentrated at the axon terminals in controls, whereas they remain distributed throughout the axon branches in Ca_V_1.1^−/−^ mice (arrows). Scale bars: 20 μm. **(c)** Labeling of synaptic vesicles and motor axons in diaphragms from control and RyR1^−/−^; DHPR^nc/nc^ mice. RyR1^−/−^; DHPR^nc/nc^ mice also display dispersed synaptic vesicles at E18.5 (arrows). Scale bar: 20 μm.

**Figure 6:**
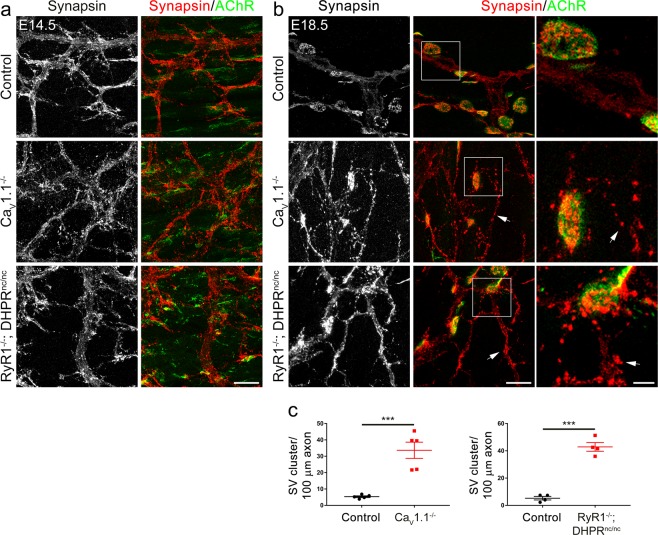
Extrasynaptic distribution of synaptic vesicles in mice lacking Ca_V_1.1 or Ca_V_1.1-mediated calcium signaling. **(a,b)** Representative micrographs of diaphragms from control, Ca_V_1.1^−/−^ and RyR1^−/−^; DHPR^nc/nc^ mice double-labeled with anti-synapsin (red) to localize synaptic vesicles and α-BTX (green) to label postsynaptic AChRs at E14.5 **(a)** and at E18.5 **(b)**. Scale bars 20 μm. Regions in white boxes in **(b)** are magnified in the micrographs on the right. Scale bar: 5 μm. Arrows in **(b)** indicate examples of ectopic synaptic vesicle clusters apart from AChR clusters. **(c)** Quantitative analysis of synaptic vesicle aggregates not correlating with postsynaptic AChR clusters at E18.5 shows significant increase in ectopic synaptic vesicle clusters per 100 μm of extrasynaptic axonal branch in Ca_V_1.1^−/−^ and RyR1^−/−^; DHPR^nc/nc^ mice compared to their control littermates. N ≥ 4 diaphragms from at least 3 litters for each genotype; mean ± SEM; t-test, ***p < 0.0001.

A hallmark of the presynaptic differentiation is the progressive accumulation of synaptic vesicles at motor nerve terminals facing postsynaptic neurotransmitter receptor clusters during innervation^22,40^. The failure of target-recognition and axon termination at the synaptic sites in the absence of Ca_V_1.1-driven calcium signaling suggested that proper presynaptic specialization might also be deficient. In order to explore a possible role of Ca_V_1.1 in this process, we labeled synaptic vesicles and motor nerve branches in diaphragms from Ca_V_1.1^−/−^ mice and their wildtype littermates at different days of fetal development using synapsin and neurofilament antibodies. At E14.5, synaptic vesicles were typically distributed all along the motor nerve branches in wildtype and mutant mice. As development proceeded, in control mice synaptic vesicles became concentrated at the nerve terminals, while extrasynaptic axonal domains increasingly became devoid of synapsin staining so that by E18.5 synaptic vesicles were essentially lacking in extra-synaptic regions (Figs. 5a, 6a,b). In Ca_V_1.1^−/−^ and RyR1^−/−^; DHPR^nc/nc^ mice this concentration of synaptic vesicles in nerve terminals failed. Even at E18.5 synaptic vesicles remained distributed throughout the nerve branches (Figs. 5, 6). This deficient concentration of synapsin-labeled vesicles in nerve terminals was equally observed in RyR1^−/−^; DHPR^nc/nc^ mice, substantiating the importance of postsynaptic Ca_V_1.1-driven calcium signaling for the differentiation of the presynaptic nerve terminals. A second synaptic vesicle marker, vesicular acetylcholine transporter (VAChT) also displayed a specific colocalization with AChR clusters in the nerve terminals in E18.5 controls diaphragms, while the extrasynaptic regions were mostly devoid of VAChT labeling. Again, this concentration of VAChT failed in Ca_V_1.1^−/−^ mice at E18.5, in which VAChT staining was present throughout the nerve branches (Suppl. Fig. 2).

**Figure 7:**
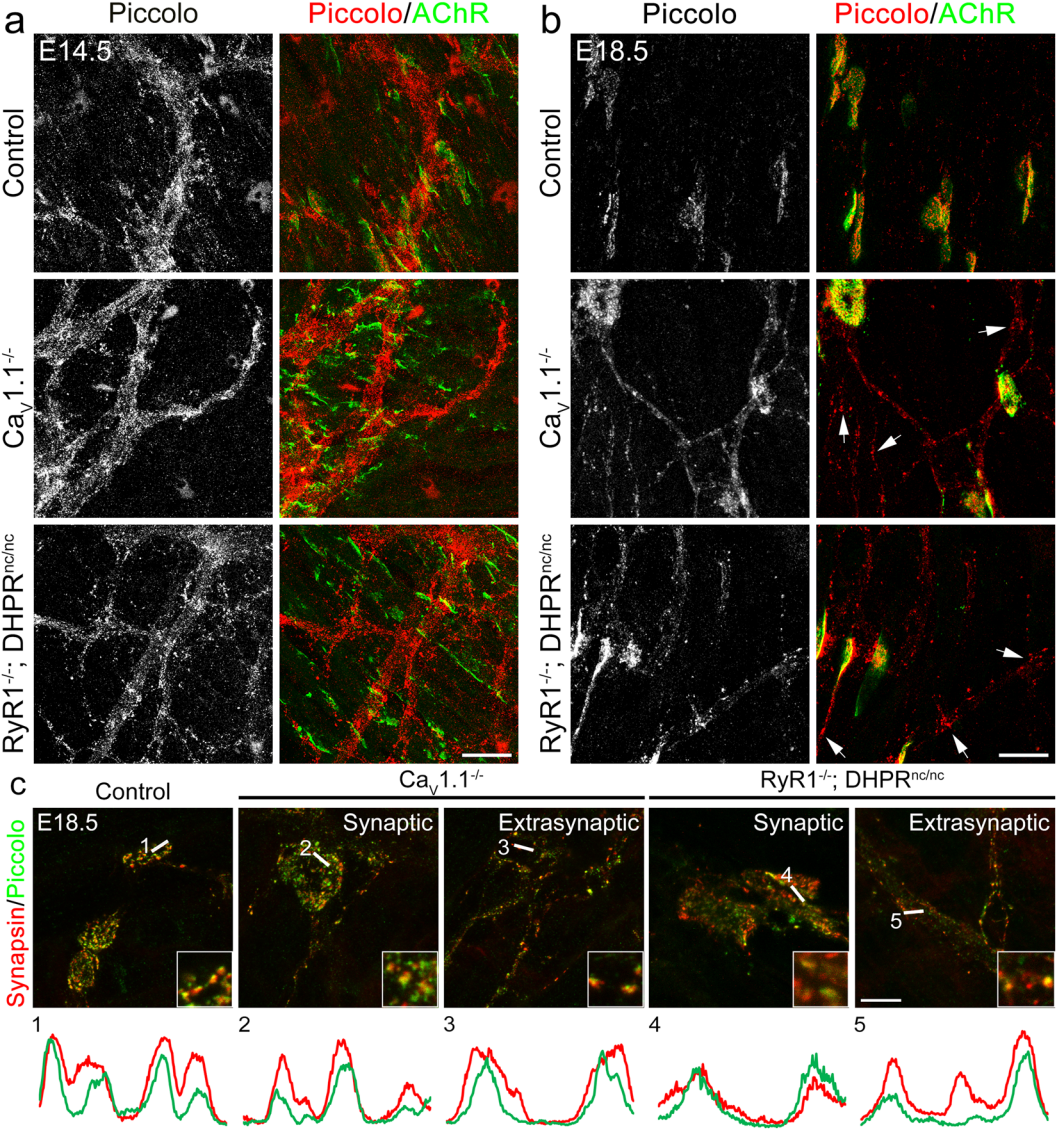
Ca_V_1.1-mediated calcium signaling controls active zone targeting to forming synapses. E14.5 **(a)** and E18.5 **(b)** diaphragms from control, Ca_V_1.1^−/−^ and RyR1^−/−^; DHPR^nc/nc^ mice are labeled with the active zone marker piccolo (red) and AChRs with α-BTX (green). At E14.5 piccolo is distributed all along the motor nerve branches, indicating that nerve terminals are not yet specialized. At this stage no difference in the distribution of the presynaptic markers was observed between the calcium channel mutants and controls. Scale bar: 20 μm. At E18.5, in control piccolo labeling became restricted to synaptic regions, whereas in mutants piccolo staining is detected also in the extrasynaptic axonal domain (examples shown by arrows). Scale bar: 20 μm. **(c)** High magnification micrographs of E18.5 diaphragms from control and mutant mice show colocalization of synaptic vesicles (red) and the active zone protein piccolo (green) in presynaptic terminals of control, Ca_V_1.1^−/−^ and RyR1^−/−^; DHPR^nc/nc^ mice, and in extrasynaptic regions in the mutant only. Linescan fluorescence intensity blots of the 4.8 μm white lines are indicated below the micrographs. Regions where line scan analysis was applied are magnified in the bottom right corner of the micrographs. Scale bar: 10 μm.

To determine whether this presynaptic defect was specific to synaptic vesicles or whether developmentally regulated accumulation of active zones was also altered in Ca_V_1.1^−/−^ and RyR1^−/−^; DHPR^nc/nc^ mice, we used a piccolo antibody along with α-BTX to label active zones and postsynaptic AChR clusters, respectively, at E14.5 and E18.5 diaphragms. Like the synapsin distribution, at E14.5 piccolo staining was observed dispersed throughout the branches of the motor axons in all three examined genotypes (Fig. 7a). At E18.5 in controls piccolo staining became restricted to the synaptic sites, whereas in Ca_V_1.1^−/−^ and RyR1^−/−^; DHPR^nc/nc^ mice, it was widely distributed throughout extrasynaptic axonal domains (Fig. 7b). Moreover, piccolo appeared to colocalize with synapsin in both synaptic and extrasynaptic regions of motor axons in Ca_V_1.1^−/−^ and RyR1^−/−^; DHPR^nc/nc^ mice (Fig. 7c). Together these data strongly indicate that aberrant branching, defasciculation, and failed target-recognition are accompanied by a lacking differentiation of presynaptic terminals of motor nerves and that all these processes, which are required for proper neuro-muscular innervation, are critically dependent on Ca_V_1.1-mediated calcium signaling in the muscle cells.

### Maintained calcium influx through embryonic Ca_V_1.1e isoform reveals a differential rescue of pre- and postsynaptic defects

**Figure 8:**
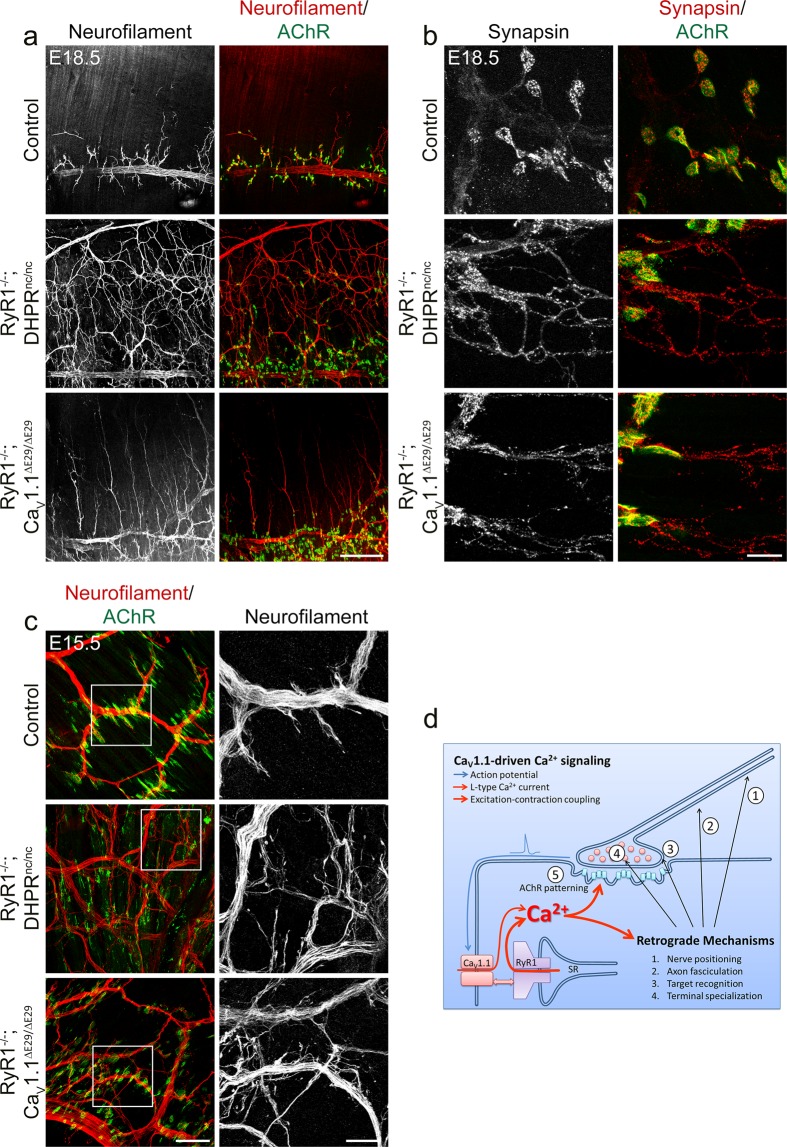
Increased calcium influx in mice expressing only the embryonic Ca_V_1.1e isoform rescues AChR patterning but not the presynaptic defects. Both double mutant mice lack RyR1, but either express a non-conducting Ca_V_1.1 (RyR1^−/−^; DHPR^nc/nc^) or a highly-conducting Ca_V_1.1e (RyR1^−/−^; Ca_V_1.1^ΔE29/ΔE29^). **(a)** Motor axons and AChRs are labeled with neurofilament antibody (red) and α-BTX (green) in left diaphragms at E18.5. RyR1^−/−^; Ca_V_1.1^ΔE29/ΔE29^ mice rescue the central AChR cluster patterning and display decreased nerve outgrowth and branching compared to RyR1^−/−^; DHPR^nc/nc^ mice. However, overshooting axon branches are still frequently observed in RyR1^−/−^; Ca_V_1.1^ΔE29/ΔE29^ mice. Scale bar: 200 μm. **(b)** Synaptic vesicles clusters (red) and AChRs (green) are labeled with synapsin antibody and α-BTX, respectively, at E18.5. Both RyR1^−/−^; DHPR^nc/nc^ and RyR1^−/−^; Ca_V_1.1^ΔE29/ΔE29^ mice display substantial synaptic vesicle staining in the extrasynaptic axonal domain. Scale bar: 20 μm. **(c)** Motor axons and AChRs are labeled with neurofilament antibody and α-BTX, respectively, in E15.5 diaphragms. Both RyR1^−/−^; DHPR^nc/nc^ and RyR1^−/−^; Ca_V_1.1^ΔE29/ΔE29^ mice display increased axon defasciculation compared to controls. Scale bar: left, 50 μm; zoomed images at right, 20 μm. **(d)** Model showing multiple roles of Ca_V_1.1-driven calcium signaling in the retrograde regulation of presynaptic differentiation at the NMJ. Action potentials initiated at the NMJ are sensed by Ca_V_1.1, which drives L-type calcium currents and SR calcium release during EC coupling in the muscle fibers. These calcium signals postsynaptically control AChR patterning (5), and presynaptically multiple aspects of motor neuron differentiation: (1) correct motor nerve positioning and projection pattern, (2) fasciculation of the motor axons, (3) target-recognition, and (4) specialization of the motor nerve terminals.

The relatively more severe branching defects in RyR1^−/−^; DHPR^nc/nc^ mice compared to Ca_V_1.1^−/−^ (see Fig. 1c), and the differences observed between homozygous RyR1^−/−^; DHPR^nc/nc^ and semi-heterozygous RyR1^−/−^; DHPR^+/nc^ mice (see Fig. 3a) suggested that the presence of a calcium leak from uncoupled RyR1 (in Ca_V_1.1^−/−^)^34^ or of an LTCC at half of the magnitude (in RyR1^−/−^; DHPR^nc/+^)^35^ ameliorated some of the presynaptic defects in the calcium channel mutants. Previously we demonstrated that abolishing the natural decline of LTCCs during late fetal development by preventing the developmentally regulated alternative splicing of Ca_V_1.1 exon 29 rescued aberrant AChR clustering in E18.5 RyR1^−/−^; Ca_V_1.1^ΔE29/ΔE29^ mice^12^. Interestingly though, excessive presynaptic growth was still observed in these double mutant mice, suggesting a differential calcium sensitivity of the mechanisms regulating pre- and postsynaptic differentiation. To substantiate these observations and to further examine whether and to what extent defects in presynaptic differentiation are maintained under conditions at which defects in AChR cluster patterning are rescued, we compared nerve branching, fasciculation, and nerve terminal differentiation in RyR1^−/−^; DHPR^nc/nc^ and RyR1^−/−^; Ca_V_1.1^ΔE29/ΔE29^ mice, both of which lack EC coupling but differ in their expression of LTCCs^31,35^. The examples in Fig. 8a show that, whereas in RyR1^−/−^; Ca_V_1.1^ΔE29/ΔE29^ mice AChR clustering is restricted to the central endplate band and the extent of excessive nerve branching is much lower than in RyR1^−/−^; DHPR^nc/nc^ mice, the secondary motor nerve branches still extend beyond of the endplate band. Moreover, in these motor nerve branches of E18.5 RyR1^−/−^; Ca_V_1.1^ΔE29/ΔE29^ diaphragm synaptic vesicles are still widely distributed like in RyR1^−/−^; DHPR^nc/nc^ mice, and fail to concentrate opposite AChR clusters as controls (Fig. 8b). These findings show that in the presence of calcium influx in RyR1^−/−^; Ca_V_1.1^ΔE29/ΔE29^ mice target recognition and nerve terminal differentiation still fail, even though the distribution of AChR clusters is fully normalized.

The striking differences in the two genotypes (RyR1^−/−^; DHPR^nc/nc^ and RyR1^−/−^; Ca_V_1.1^ΔE29/ΔE29^) between pre- and postsynaptic deficiencies can already be observed at E15.5. Figure 8c shows that the size and distribution of AChR clusters in the RyR1^−/−^; Ca_V_1.1^ΔE29/ΔE29^ diaphragms resemble those of the wildtype controls; whereas the excessive axonal branching, defasciculation, growth beyond the AChR clusters, and the presence of growth cone-like structures all resemble the deficiencies in the RyR1^−/−^; DHPR^nc/nc^ mice. Thus, the observed partial rescue by increasing the LTCCs during late fetal development supports the dependence of motor nerve differentiation on muscle calcium signals, while the differential effects on AChR clustering and motor axon growth and differentiation demonstrates that proper presynaptic differentiation requires stronger calcium signals than postsynaptic differentiation.

## Discussion

This study focuses on the role of Ca_V_1.1-dependent calcium signaling in regulating the presynaptic motor neuron differentiation during NMJ formation. By analyzing two distinct mouse models, both of which lack activity-dependent postsynaptic calcium signaling, we report a central role of muscle calcium signals in the regulation of multiple distinct aspects of the presynaptic differentiation at the forming NMJ. The deficiencies displayed by mice lacking muscle calcium include: (1) the presence of an additional ectopically located nerve trunk as well as excessive branching and aberrant projections of secondary branches from these ectopically located trunks, (2) unrestricted defasciculation of the motor nerves, (3) the failure of axon navigation and target recognition, and (4) the failure of correct accumulation of synaptic vesicles and active zone markers at the nerve terminals.

These presynaptic defects accompany defects in the patterning of postsynaptic AChR clusters reported previously in the same genetic mouse models^12^. The observation of similar NMJ phenotypes in Ca_V_1.1^−/−^ and in RyR1^−/−^; DHPR^nc/nc^ demonstrates that Ca_V_1.1-driven calcium signals, but not the absence of one or the other calcium channel protein is causal for these presynaptic deficiencies. The somewhat greater severity of the deficiencies in the RyR1^−/−^; DHPR^nc/nc^ mice compared to Ca_V_1.1^−/−^ mice can be explained by the total absence of calcium influx and release in the double knockout, while calcium leak through the uncoupled RyR1^34^ may mitigate the phenotype in Ca_V_1.1^−/−^ mice. Interestingly, a semi-heterozygous genotype (RyR1^−/−^; DHPR^+/nc^) with only half of the calcium influx^35^ is capable of largely rescuing the AChR patterning defect but not the motor nerve defects, indicating that the mechanisms regulating presynaptic differentiation require more robust muscle calcium signals than the mechanism regulating AChR patterning. Along these lines the continued expression of the highly-conducting embryonic Ca_V_1.1e isoform in RyR1^−/−^; Ca_V_1.1^ΔE29/ΔE29^ mice^12,31^, a condition that rescues AChR patterning defects occurring in RyR1^−/−^ mice at E18.5, was also unable to rescue the presynaptic defects (Fig. 8a,b). Ca_V_1.1 and RyR1 are both skeletal muscle -specific channels, specialized for their cooperate function in skeletal muscle EC coupling, and their null-mutants display the typical perinatally lethal skeletal muscle phenotype. To our knowledge neither Ca_V_1.1 nor RyR1 is expressed in motor neurons. If one or the other would be expressed in motor neurons, the presynaptic phenotype should be selective for the corresponding mouse model, but normal in the respective other. In the lack of any such evidence, it is very unlikely that the motor nerve defects reported here resulted directly from presynaptic mechanisms. Rather, our observations indicate that skeletal muscle calcium signals are crucial for the regulation of the motor nerve differentiation, that the severity of these presynaptic defects inversely correlates with the magnitude of the calcium signals remaining in the various genotypes, and that activating the trans-synaptic signaling mechanisms requires stronger calcium signals than the previously reported calcium-dependent regulation of AChR patterning.

Distinct calcium sensitivities are indicative of distinct signaling mechanisms for the pre- and postsynaptic differentiation downstream of calcium. Importantly, several lines of evidence show that the presynaptic deficiencies are not simply the consequence of the postsynaptic AChR patterning defects. Positioning of the motor nerve trunk, axon fasciculation, branching, and target-recognition occur apart and independently from the central patterning of MuSK and AChR clusters^2,14,15,20,21^. Most importantly, in the calcium channel mutants motor axons appear to ignore AChR clusters and grow well beyond them, indicating that their excessive outgrowth and lack of differentiation are independent of the distribution of their target structures. Similarly, direct effects of mechanisms upstream of calcium, like ACh release and muscle activity can be excluded, because spontaneous and nerve-induced electrical activity are intact in DHPR-deficient mice^33,41^, and likely also in RyR1^−/−^; DHPR^nc/nc^ mice. Together, these findings demonstrate a key role of activity-dependent calcium signaling mediated by Ca_V_1.1 in the postsynaptic signaling pathways which regulate the growth and differentiation of the motor axon independently of calcium´s demonstrated role in clustering and patterning of AChRs^12,33^.

In the past, similar presynaptic defects have been described in various mouse models presenting either synaptic transmission defects or defects in postsynaptic differentiation. For example, axon sprouting and growth beyond endplates have been observed in mouse models lacking acetylcholine release^10,42,43^ or expressing inactive AChRs^24^. Also, nerve sprouting defects consistently have been observed in denervated and pharmacologically inactivated muscles (for review see^44,45^). In light of our current findings the presynaptic phenotype in these mice could be explained by the lack of retrograde mechanisms regulated by activity-dependent calcium signaling in the muscle cells. In other mouse models resulting in the total lack of AChR clusters (such as rapsyn, agrin, MuSK, LRP4, Dok-7 mutants)^6,18,19,46,47^ excessive neurite outgrowth has commonly been interpreted as the consequence of the lacking targets. Motor axons continue to grow while unsuccessfully searching for their postsynaptic target structures. Considering that, as a consequence of missing synapses also activity-dependent calcium signaling will be compromised, this indirect pathway may be a likely alternative explanation for the overgrowth of axons in mice lacking AChR clustering. Consistent with this notion, axon growth is arrested upon synapse formation in muscles overexpressing MuSK, while AChRs are clustered in a wider region than the innervation territory observed by Kim and Burden^2^.

Our current findings suggest that during fetal development Ca_V_1.1 acting as L-type calcium channel and as voltage-sensor in EC coupling translates the muscle electrical activity into calcium signals which regulate the expression and/or display of trans-synaptic cues, which in turn retrogradely regulate motor nerve and pre-synapse differentiation (Fig. 8d). Several muscle-derived retrograde signals acting during NMJ development have been identified^13^, and one or more of these might be regulated by activity and Ca_V_1.1-dependent calcium signaling. Strikingly, the NMJ phenotype of genetic models for some of these putative retrograde signals closely resemble specific aspects of the phenotype reported here. For example, β-catenin/Slit2/Robo pathway controls proper fasciculation of the motor nerves as in mice lacking Slit2/Robo^15^ or muscle β-catenin^14^ the nerve trunk is mislocated in the muscle periphery, as observed here in the calcium channel mutant mice. Nevertheless, in mouse models for this pathway motor axons still recognize their target structures and do not overshoot AChR clusters, and synaptic vesicles appear to be correctly localized in the axon terminals^14–16^. On the other hand, muscle-specific knockout of LRP4^20^ or β1-integrin^17^ results in a failure of target-recognition by the motor axons. Like in the calcium channel mutants reported here, axons fail to stop at the AChR clusters. However, in these mice, a mislocalized nerve trunk or failed synaptic vesicle targeting were not reported. Finally, in FGFR2b-null^22^ and NCAM-null^23^ mice, synaptic vesicles are aberrantly distributed throughout the periterminal axon branches, while these mice do not show any defects of fasciculation, axon guidance, termination or branching.

Combined these studies indicate that positioning and fasciculation of the nerve branches, outgrowth and target recognition, and the differentiation of presynaptic nerve terminals are separable processes, each regulated by a specific retrograde signaling cascade. Our observation that all of these features fail in Ca_V_1.1^−/−^ and RyR1^−/−^; DHPR^nc/nc^ mice strongly suggests that Ca_V_1.1-driven calcium signals not only regulate but also orchestrate multiple downstream signaling mechanisms involved in the correct presynaptic differentiation of the motor axons throughout the embryonic NMJ development (Fig. 8d).

## Materials and Methods

### Animals

*Mice*. Ca_v_1.1^−/−^^25^, RyR1^−/−^; DHPR^nc/nc^^12,35–37^ and RyR1^−/−^; Ca_V_1.1^ΔE29/ΔE29^^12,31,36,37^ mice have been described previously. Skeletal muscle of homozygous Ca_V_1.1^−/−^ mice lacks L-type calcium currents and EC-coupling^25^; however uncoupled RyRs are still expressed in triad junctions and cause a calcium leak from SR stores^34^. Skeletal muscle of homozygous RyR^−/−^ mice lacks EC coupling^36,37^; however Ca_V_1.1 is still expressed in the triads and particularly the embryonic splice variant can support depolarization-induced calcium influx^31^. DHPR^nc/nc^ mice carry a point mutation which causes the complete ablation of L-type calcium currents through the DHPR, yet does not affect its coupling with RyR1 and activity-induced calcium release^35^. Ca_V_1.1^ΔE29/ΔE29^ mice exclusively express the embryonic Ca_V_1.1e splice variant, which, like the adult Ca_V_1.1a variant, functions as voltage-sensor in EC coupling, but in addition conducts sizable L-type calcium currents at physiological membrane potentials^31^.

Homozygous mutant mice for Ca_v_1.1^−/−^ and their control littermates were obtained from heterozygous matings. RyR1^−/−^; DHPR^nc/nc^, RyR1^−/−^; DHPR^+/nc^ mice and their control littermates were obtained by RyR1^+/−^; DHPR^+/nc^ x RyR1^+/−^; DHPR^nc/nc^ matings. RyR1^−/−^; Ca_V_1.1^ΔE29/ΔE29^ and their control littermates were obtained by RyR1^+/−^; Ca_V_1.1^ΔE29/ΔE29^ matings. Sperm plugs were checked daily at 8:00 am and 5:30 pm. The day a plug was detected was counted as embryonic day E0.5. Embryos were collected at the selected days of pregnancy by cesarean section of sacrificed pregnant mice. All experimental protocols conformed to the guidelines of the European Community (86/609/EEC) and were approved by the Austrian Ministry of Science (BMWFW-66.011/0002-WF/V/3b/2015).

### Genotypings

Tissue samples from embryos were lysed in 50 μl of HotShot Lysis buffer at 95 °C for at least 30 minutes then neutralized by adding 50 μl of HotShot Neutralize Buffer as described previously^48^ and used for PCR genotyping and gel electrophoresis. The following primers were used (purchased from *Eurofins*):

Ca_v_1.1^−/−^ mice, forward: GGCATGCAGATGTTCGGGAAGATC, reverse: GCAGCTTTCCACTCAGGAGGGATCCAGTGT. PCR product (270 bp) was cut with EarI (New England Biolab, R05285). The wildtype allele yielded 100 bp and 170 bp fragments, the knockout allele yielded a 270 bp fragment.

RyR1^−/−^ mice, forward: GGACTGGCAAGAGGACCGGAGC, reverse WT: GGAAGCCAGGGCTGCAGGTGAGC and reverse KO: CCTGAAGAACGAGATCAGCAGCCTCTGTCCC. The wildtype allele yielded a 400 bp fragment, the knockout allele yielded a 300 bp fragment.

DHPR^nc/nc^ mice, forward: CTAAGCCTACTCACACCTTGATAACAT, reverse: GAGAGGACAGTTTCTTGCCAGACCTACACCCCTTGG. PCR product (710 bp) was cut with Pf1FI (New England Biolab, R05955). The wildtype allele yielded a 710 bp fragment, the knockout allele yielded 529 bp and 181 bp fragments.

Ca_V_1.1^ΔE29/ΔE29^ mice, forward: CCTGTCTCTGTCTGGTCTTCC reverse: GCCTGCTCTAAGGAAAGGGA. The wildtype allele yielded a 373 bp fragment, the knockout allele yielded a 344 bp fragment.

### Methods details

#### Immunohistochemistry and Image processing

The middle part of the embryos containing the ribcage, liver, diaphragm and lungs was fixed with 4% paraformaldehyde in 0.1 M phosphate buffer (pH: 7.2) for 1 hour at room temperature. Subsequently diaphragms were dissected in PBS and incubated in 0.1 M glycine in PBS for 1 h at room temperature, permeabilized and blocked in PBS containing 1% bovine serum albumin (BSA), 5% normal goat serum (NGS) and 0.2% Triton X-100 overnight at 4 °C. Primary antibodies for rbSynapsin (1/10,000, Synaptic System), mSynapsin (1/1,000, Synaptic System), rbNeurofilament-200 (1/1,000, Sigma), rbPiccolo (1/1,000, Synaptic System), rbVAChT (1/5,000, Synaptic System) were applied at 4 °C overnight. The muscle samples were washed three times at 1 h intervals and incubated with anti-rabbit Alexa594 (1/4,000), anti-rabbit Alexa488 (1/4,000), anti-mouse Alexa594 (1/4,000) and/or α-BTX conjugated with Alexa488 (1/8,000, all from Invitrogen) for 2 h at room temperature. After extensive washing, diaphragms were flat-mounted in *Vectashield*. Images were captured on a Leica microsystems SP5 laser scanning confocal microscopy using LasAF acquisition software (Leica microsystems). Fluorescence was excited using the 488 nm and 561 nm laser lines and recorded at a bandwidth of 493–556 nm (green channel) and 566–752 nm (red channel), respectively. 8-bit images with 1024 × 1024 pixels were acquired at 400 Hz scan speed. Maximum projections of acquired z-serial images were generated and analyzed using *Metamorph* image processing software. Post-imaging processing and arrangement of the images were performed with *Adobe Photoshop CS6*. Only linear adjustments were applied to correct brightness and contrast.

### Quantification and statistical analysis

#### Ectopic neurite length analysis

Tile scan images of the left dorsal diaphragms were taken using a 40X oil objective with SP5 laser scanning confocal microscopy. Using *Metamorph* software, the outer border of the endplate band was traced. Motor axon branches which crossed that border and ended outside the endplate band were traced back from the axon tip to the endplate band to measure the ectopic neurite length.

#### Overshooting axon analysis

Images from the left ventral diaphragm were captured using the SP5 laser scanning confocal microscope with a 20X objective. A line along the border of the endplate band was drawn and axons crossing this line were manually counted using *Metamorph* software. The length of the endplate band and number of the axons crossing this line were used to calculate number of overshooting motor axons per 100 μm of endplate band.

#### Extrasynaptic axon ending analysis

Images of the left diaphragm stained with rbNeurofilament antibody and α-BTX were captured with an SP5 laser scanning confocal microscope using a 63X objective. The number of axon endings that did not correlate with postsynaptic AChR patches were manually counted using *Metamorph* software. The total area of the frame (246 × 246 μm) was used to calculate extrasynaptic axon ending count per frame.

#### Synaptic vesicles analysis in axons

Images of the left diaphragm stained with rbSynapsin and α-BTX were captured with an SP5 laser scanning confocal microscope using a 63X objective. For each image, the number of synaptic vesicle clusters that did not correlate with postsynaptic AChR clusters were manually counted using *Metamorph* software. The length of motor axon branches in the image was measured and number of synaptic vesicle clusters per 100 μm of motor axon branch was calculated.

#### Statistical analysis

Student t-test was applied to assess statistical significance of differences between two groups. One-way ANOVA with Tukey’s multiple comparison test was applied to assess statistical significance of differences between three groups. For all analysis GraphPad Prism was used.

## Supplementary information

Supplementary File
