# Supplementary material for: Postsynaptic CaV1.1-driven calcium signaling coordinates presynaptic differentiation at the developing neuromuscular junction
Source: Sci Rep. 2019 Dec 5;9:18450. doi: 10.1038/s41598-019-54900-w (PMC6895222; doi:10.1038/s41598-019-54900-w)

Supplumentary Information for

Postsynaptic $Ca_V1.1$-driven calcium signaling coordinates presynaptic differentiation at the developing neuromuscular junction

Mehmet Mahsum Kaplan & Bernhard E. Flucher

Department of Physiology and Medical Physics, Medical University Innsbruck, 6020 Innsbruck, Austria

To whom correspondence should be addressed:

Bernhard E. Flucher, PhD
Department of Physiology and Medical Physics, Medical University Innsbruck
Schöpfstraße 41, A-6020 Innsbruck, Austria
Phone: +43 512 9003 70836, Email: bernhard.e.flucher@i-med.ac.at

This file includes Supplementary Figure 1 and 2.

Supplementary Figure Legend

Suppl. Fig. 1: Aberrant motor nerve defasciculation in $Ca_V1.1^{-/-}$ and $RyR1^{-/-}$; $DHPR^{nc/nc}$ E14.5 diaphragms. Motor axons and AChRs are labeled with neurofilament antibody (red) and α-BTX (green). Motor nerves in diaphragm of control mice at E14.5 run in fascicles with small branches projecting towards the AChR clusters. In $Ca_V1.1^{-/-}$ and $RyR1^{-/-}$; $DHPR^{nc/nc}$ diaphragms motor nerves are severely defasciculated and show excessive branching. Scale bar: 50 µm. Motor axons in white boxes are displayed in zoomed images on the right. Scale bar: 20 µm.

Suppl. Fig. 2: Extrasynaptic localization of VAChT in $Ca_V1.1^{-/-}$ diaphragms. Vesicular acetylcholine transporter VAChT is stained together with AChRs in E18.5 diaphragms of $Ca_V1.1^{-/-}$ mice and their control littermates. Similar to synapsin (Fig. 6), VAChT is colocalized with AChR clusters and absent from extrasynaptic regions in controls, but in $Ca_V1.1^{-/-}$ displays ectopically located clusters throughout the neurites (shown by arrows). Scale bars 20 µm. Regions in white boxes are magnified in the micrographs on the right. Scale bar: 5 µm.

Supplementary Figure 1

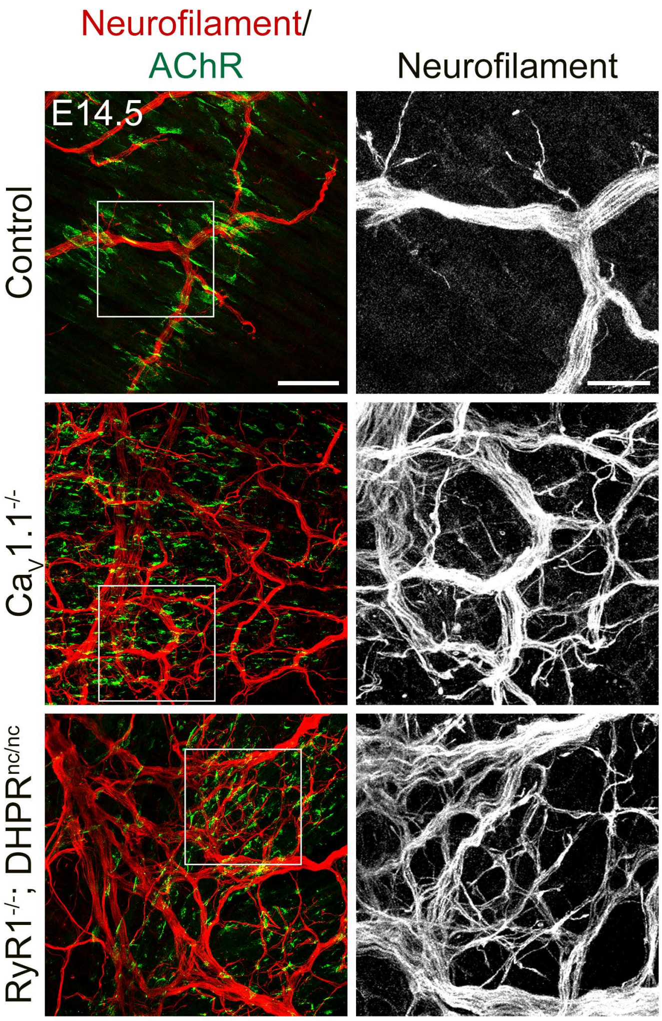

Supplementary Figure 2

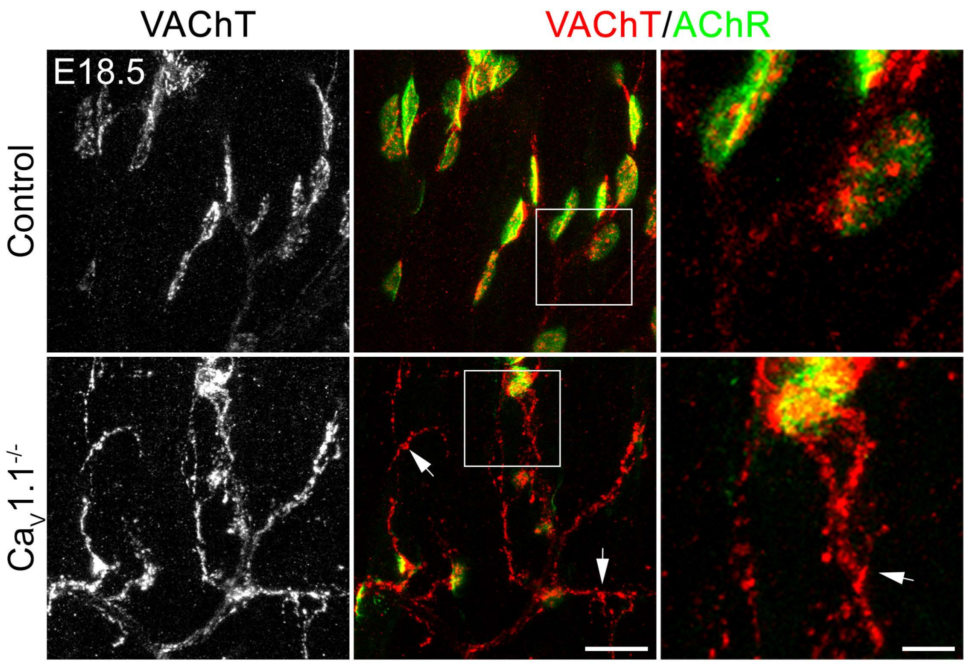

Supplement: Supplementary file 1 — Supplementary File [file 41598_2019_54900_MOESM1_ESM.pdf]
